# The regulatory role of histidine kinase in modulating nucleoside metabolites in *Streptomyces noursei* CK-15

**DOI:** 10.3389/fmicb.2026.1692886

**Published:** 2026-04-01

**Authors:** Yutong Liao, Jiabei Xie, Qingqing Zhao, Hua Yu, Beibei Ge

**Affiliations:** State Key Laboratory for Biology of Plant Diseases and Insect Pests, Institute of Plant Protection, Chinese Academy of Agricultural Sciences, Beijing, China

**Keywords:** biosynthesis, synergistic effect, gene clusters, Toyocamycin, Wuyiencin

## Abstract

Histidine kinase (HK), a vital component of the two-component system (TCS), regulates the synthesis of secondary metabolites and is integral to key processes related to the growth and environmental adaptation of *Streptomyces*. The *SHK* gene may govern the manufacture of the nucleoside-type secondary metabolites Toyocamycin and Wuyiencin. This study generated a histidine kinase overexpression mutant strain (*S. noursei oSHK*) exploiting Δ*toyG* transcription data. This mutant strain was seen to suppress the growth of the CK-15 strain, resulting in a 25.94% reduction in Wuyiencin levels and a 17.64% decrease in Toyocamycin levels, while also decreasing spore formation and hyphal development rate. The SHK gene demonstrates reduced efficacy against bacteria and compromised nitrogen use. These findings suggest a detrimental regulatory influence of the *SHK* gene on strain viability and secondary metabolite synthesis in *S. noursei* CK-15, and this study provides theoretical justification for the modification of high-yielding Wuyiencin strains.

## 1 Introduction

In agriculture, secondary metabolites produced by *Streptomyces* are widely utilized due to their diverse agricultural values, including biocontrol of plant pathogens and promotion of crop growth. They serve as eco-friendly biological pesticides by inhibiting the growth of pathogenic fungi: for instance, *Streptomyces purpeofuscus* CC2-6 secretes proteases, cellulases, and chitinases to mitigate soil-borne diseases (Wang et al., 2024), while *Streptomyces corchorusii* CG-G2 emits volatile organic compounds with antifungal activities (Li et al., 2024). Beyond Streptomyces, Bacillus species have emerged as another class of high-potential biocontrol agents with multifunctional advantages—Bacillus mojavensis KRS009, isolated from the rhizosphere of healthy cotton in pathogen-infested fields, not only effectively suppresses cotton Verticillium wilt by inducing salicylic acid and jasmonic acid-dependent immune pathways but also enhances plant salt tolerance and promotes growth under saline stress (Zhao et al., 2024). This further confirms that beneficial microorganisms and their metabolites are core to sustainable agricultural practices. Besides biocontrol, Streptomyces-derived secondary metabolites can also regulate crop growth and enhance stress resistance—decoyinine, for example, exhibits significant growth-promoting effects in rice and Arabidopsis, potentially increasing rice yield by 6.2%−19.6% (Yang et al., 2023; Liu et al., 2022; Shi et al., 2022), and further improves soil conditions to facilitate nutrient absorption.

In line with the irreplaceable role of plant protection in improving agricultural ecosystem productivity, alleviating food insecurity, and meeting the exponentially growing food demand, the new era of plant protection urgently requires innovative technologies that adhere to the principles of health, environmental friendliness, and sustainable development (Chen and Wu, 2024). This underscores the significance of investigating the regulatory mechanisms of Streptomyces-derived secondary metabolites: such research not only provides a theoretical basis for optimizing the yield of bio-pesticides and advancing the green control of plant diseases but also supports the full exploitation of their multi-faceted values in sustainable agriculture.

Two-component systems (TCSs) are the principal mediators of signal transduction commonly found in *Streptomyces*. They assist cells in seeing, responding to, and adapting to diverse environmental situations (Papon and Stock, 2019). The production of *Streptomyces* secondary metabolites is meticulously governed by the two-component regulatory system (TCS) (Dikiy et al., 2023). The N-terminal sensor domain of histidine kinases (HK) identifies exterior signals and relays them via the transmembrane domain to the kinase domain inside the cell. The dimerization and histidine phosphorylation regions inside the C-terminal kinase domain facilitate the self-phosphorylation of histidine kinase (HK), therefore modulating the expression of downstream gene clusters associated with secondary metabolism. This incorporates external cues to accurately modulate *Streptomyces* physiological processes and activate intracellular response mechanisms (Zschiedrich et al., 2016). It has been reported that in *Streptomyces lividans*, the regulation of environmental phosphate metabolism is mediated by the two-component signal transduction system PhoR-PhoP, and its disruption leads to the overexpression of the secondary metabolites actinorhodin and undecylprodigiosin (Zhu, 2008). In *Streptomyces coelicolor*, the novel two-component system DraR-K perceives environmental signals (high-concentration nitrogen sources), differentially regulates the biosynthesis of various antibiotics, and affects aerial mycelia and spores (Yu et al., 2012). Additionally, *S. coelicolor* harbors a key two-component signal transduction system AfsQ1/Q2, which consists of the histidine kinase AfsQ1 and the response regulator AfsQ2. Its core function is to integrate nutritional metabolic signals such as carbon, phosphorus, and nitrogen, participate in the metabolic regulation of these nutrients, and simultaneously coordinate the biosynthesis of multiple antibiotics and the morphological development of the strain to maintain the balance between primary and secondary metabolism (Bednarz et al., 2019).

In conclusion, histidine kinase (HK) functions as a sensor in two-component signaling systems (TCS) and is essential for controlling the production of secondary metabolites in *Streptomyces*. A mutant strain of the orphan histidine kinase OhkA from *S. coelicolor* exhibits decreased aerial mycelium and spore development on mannitol-soybean meal (MS) medium, however overproduces several antibiotics, including actinomycin D and calcium-dependent antibiotics (Zheng et al., 2021). Consequently, the examination of histidine kinases can elucidate the molecular regulatory network that controls secondary metabolite synthesis in *Streptomyces*, offering specific targets for genetic engineering aimed at modifying strains and enhancing the efficiency of secondary metabolite synthesis.

Contemporary research methodologies for histidine kinases encompass molecular biology, biochemistry, structural biology, and fermentation engineering technologies. This research will utilize molecular biology techniques to examine the role of histidine kinases in *Streptomyces noursei* CK-15. Our team earlier developed a Δ*toyG* mutant strain and observed alterations in the synthesis of Toyocamycin and Wuyiencin in the fermentation broth of the mutant strain (Liu, 2020). Toyocamycin and Wuyiencin are nucleoside-type secondary metabolites, and it is postulated that their synthesis is regulated by histidine kinase. Consequently, by analyzing transcriptome data from the Δ*toyG* mutant and the wild-type CK-15, we seek to pinpoint the critical target areas affecting the production of both molecules. This work found histidine kinase genes based on the transcriptional data of Δ*toyG*. Histidine kinase has regulatory influences on secondary metabolites. Consequently, a mutant strain with overexpression of the histidine kinase gene (*SHK*) was developed to elucidate its effects on the CK-15 strain, offering theoretical guidance for the targeted modification of transgenic organisms producing Wuyiencin.

## 2 Materials and methods

### 2.1 Test strains

*S. noursei* CK-15 was screened and preserved by the Agricultural Antibiotic Research Group of the Institute of Plant Protection, Chinese Academy of Agricultural Sciences; the *toyG* deletion mutant strain was constructed and preserved by our laboratory; the indicator organism for bioactivity testing of fermentation broth, *Rhodotorula rubra* (Zeng et al., 1995), pSETC is a single-copy integrative shuttle plasmid constructed in our laboratory by modifying the vector pSET152 (Flett et al., 1997). It carries the strong constitutive promoter PSF14 (derived from *Streptomyces fradiae*, which drives high-level gene expression in *Streptomyces*) (Li et al., 2007) and the apramycin resistance gene (apr) for selection. The donor strain *Escherichia coli* ET12567 (pUZ8002) was harboring this plasmid was also obtained from our laboratory. and harbors the helper plasmid pUZ8002. pUZ8002 is a traG-deficient derivative of pRK2, carrying the chloramphenicol resistance gene (cat) to maintain the plasmid in *E. coli* (Zeng et al., 1995).

### 2.2 Test reagents and culture media

The bacterial cells grow rapidly in seed medium (glucose 20.0 g/L, peptone 6.0 g/L, yeast extract 6.0 g/L, NaCl 10.0 g/L). *Escherichia coli* DH5α was used as cloning host, respectively. At 37 °C, *E. coli* strains were cultured in LB medium containing 10 g/L tryptophan, 5 g/L yeast extract, and 10 g/L NaCl (Liu et al., 2024). 2 × YT (yeast extract 10.0 g/L, peptone 16.0 g/L, NaCl 3.0 g/L) medium was used for conjugation transfer. MS medium (soybean flour 20.0 g/L, peptone 20.0 g/L, mannitol 20.0 g/L, agar 17.0 g/L) can be used for normal cultivation and conjugation transfer of *Streptomyces*. Wuyiencin fermentation medium (cake flour 20.0 g/L, (NH_4_)_2_SO_4_ 4.0 g/L, glucose 20.0 g/L, corn flour 30.0 g/L, CaCO_3_ 3.0 g/L) and Toyocamycin fermentation medium (glucose 5.0 g/L, yeast extract 5.0 g/L, peptone 5.0 g/L, beef extract 5.0 g/L, corn flour 4.0 g/L, soybean cake flour 10.0 g/L, starch 20.0 g/L, cobalt chloride 0.02 g/L, calcium carbonate 4.0 g/L) were used to prepare Wuyiencin fermentation broth and Toyocamycin fermentation broth, respectively. M3G medium (NH_4_)_2_SO_4_ 10.0 g/L, glucose 50.0 g/L, yeast powder 5.0 g/L, KH_2_PO_4_ 1.4 g/L, K_2_PO_4_·3H_2_O 0.8 g/L, MgSO_4_·7H_2_O 0.5 g/L, ZnSO_4_·7H_2_O 0.04 g/L, FeSO_4_·7H_2_O 0.03 g/L.

### 2.3 Transcriptome sequencing and analysis

Wild-type strain CK-15 and mutant strain Δ*toyG* spores were separately inoculated into seed medium and cultured at 220 rpm and 28 °C for 24 h. The cells were then collected,and total RNA was extracted from each strain using the Ultrapure RNA Kit (Cwbio,Beijing,China). Subsequent cDNA library construction and Illumina second-generation high-throughput sequencing were performed by Beijing Aovisun Technology Co., Ltd. Transcriptome information analysis workflow: first, raw data were obtained from the sequencer and quality assessed using tools such as FastQC. Next, HISAT2 and other software were used to align the data with the reference genome/transcriptome, quantify gene expression using metrics such as FPKM and TPM, and validate sample reliability through correlation analysis to screen for differentially expressed genes. Finally, GO enrichment (analyzing function, location, etc.) and KEGG enrichment (exploring pathway mechanisms) were performed on the differentially expressed genes to deeply explore transcriptional regulation and molecular response patterns, providing key information for studying the molecular mechanisms of biological activities.

### 2.4 Screening and functional enrichment analysis of differentially expressed genes

Gene expression levels were analyzed for each sample using HTSeq software (Putri et al., 2022) with the union model. In addition, raw counts between wild-type and mutant strains were standardized, and then statistical models were used to calculate the probability of hypothesis testing (*P*-value). Finally, multiple hypothesis testing correction (BH) was performed to obtain the false discovery rate (FDR, with *P*adj being its common form), thereby completing the differential expression analysis and screening for genes with significant differences between different samples (the threshold for differential gene screening is generally: |log2(FoldChange)| > 1 and *P*adj < 0.05). The statistically significant differentially expressed genes were annotated using GO (Gene Ontology, https://www.geneontology.org/), KEGG (Kyoto Encyclopedia of Genes and Genomes, https://www.kegg.jp), and the NCBI non-redundant protein sequence database (https://ftp.ncbi.nlm.nih.gov/blast/db/) to perform enrichment analysis on the differentially expressed genes and obtain visual results.

### 2.5 Recombinant plasmid construction

Construction of the *SHK* gene overexpression plasmid: Using the *S. nuorsei* CK-15 genomic DNA as a template, the histidine kinase gene (*SHK*) was amplified using primers *SHK*-F/R PCR Thermocycler (Tiangen Biotech Co., Ltd., Beijing, Beijing, China). The plasmid pSETC was double-digested with *Bam*H I and *Spe* I restriction enzymes to prepare a linearized vector. The linearized pSETC plasmid was then assembled with the *SHK* fragment using the Gibson Assembly one-step isothermal method. The recombinant plasmid was obtained.

### 2.6 Construction and screening of overexpressed strains

The recombinant plasmid pSTEC-*SHK* was chemically transformed into *E. coli* ET12567, then introduced into *Streptomyces* CK-15 via conjugative transfer (Tang et al., 2017). The cells were cultured at 30 °C for 18 h, supplemented with ampicillin and naphthoquinone acid, and further cultured at 30 °C for 5 days to isolate conjugates for genomic DNA extraction. Since the plasmid vector pSETC is an integrative shuttle plasmid, it is transferred into *Streptomyces* CK-15 via conjugation, after which it integrates into the *Streptomyces* genomic DNA. Free plasmids cannot survive independently (Flett et al., 1997). The resistance gene in the plasmid serves as a marker gene for screening recombinant strains, and its presence is verified using primers APR-F/APR-R. This is the method used in the conjugation transfer approach to confirm whether the recombinant expression plasmid has been successfully transformed (Shen et al., 2009; Nie et al., 2001). The recombinant strains are grown in antibiotic-free medium for two generations until sufficient spore production is achieved, and well-growing strains are selected and preserved for subsequent use (Li et al., 2007).

### 2.7 Validation of SHK gene expression in overexpressed strains by quantitative real-time PCR (qPCR)

Quantitative real-time PCR (qPCR) was performed to determine the relative expression level of the SHK gene in the overexpressed strain oSHK: mycelial blocks of *S. noursei* CK-15 (wild-type strain) and oSHK (SHK-overexpressed strain) were first inoculated into seed medium and incubated at 28 °C with shaking at 220 rpm for 48 h, after which a 2 ml aliquot of the seed culture was centrifuged at 12,000 × g for 20 min to harvest the mycelium. Total RNA was then isolated from the collected mycelium following the instructions of the TransZol Up Simple RNA Kit (TransGen Biotech, China), and the purity and concentration of the extracted RNA were determined using a NanoDrop 2000 spectrophotometer (Thermo Fisher Scientific, USA)—RNA purity was considered qualified if the A260/A280 ratio ranged from 1.8 to 2.0, and the RNA concentration of each sample was required to be ≥50 ng/μl, while RNA integrity was verified by 1% agarose gel electrophoresis with clear 23S rRNA and 16S rRNA bands (without obvious degradation) indicating acceptable integrity. Genomic DNA removal and first-strand cDNA synthesis were synchronously conducted using the TransScript All-in-One First-Strand cDNA Synthesis SuperMix for qPCR (TransGen Biotech, China), and the synthesized cDNA was diluted 10-fold with RNase-Free dH_2_O before being stored at −20 °C for subsequent use. The cDNA product was further diluted 1.5-fold to serve as the template for qPCR, with amplification carried out using the ArtiCanCEO SYBR qPCR Mix (Tsingke Biotechnology, China) and the detailed reaction system provided in Supplementary Table S3; the qPCR program was set as follows: pre-denaturation at 95 °C for 30 s, followed by 40 cycles of denaturation at 95 °C for 5 s and annealing/extension at 60 °C for 30 s, and finally melting curve analysis (65–95 °C with a temperature increment of 0.5 °C every 1 s) to verify primer specificity, which was confirmed by a single melting peak without primer dimer formation. The relative expression level of the SHK gene was calculated using the 2^−^ΔΔCt method assuming an amplification efficiency of 100%, with the 16S rRNA gene used as the internal reference to calibrate and normalize the SHK gene expression level, and the wild-type CK-15 strain serving as the calibrator (set to 1) to determine the relative SHK gene expression level in the oSHK strain through comparison.

### 2.8 Preliminary identification of target gene function

#### 2.8.1 Analysis of culture characteristics of strains with overexpression of target genes

CK-15 wild-type strain and target gene overexpression strain *oSHK* were streaked onto MS medium and incubated at 28 °C. Observations were made every 24 h for a total of 168 h.

#### 2.8.2 Cell growth analysis of target gene overexpression strains

Add CK-15 wild-type strain and *oSHK* overexpressing strain blocks to 100 ml seed medium, set the shaking incubator parameters to 28 °C and 220 rpm (Shanghai Zhichu Instrument Co., Ltd., Shanghai, China), and measure the Optical Density 600 nm value of the seed solution after 1 day of cultivation. Add 2 ml of CK-15 seed solution to 100 ml M3G medium, and add seed solutions of each overexpressing strain at the corresponding ratios, with three replicates per treatment. Samples were collected every 6 h, totaling 16 samples (over 90 h). First, filter paper dried to a constant weight was used for filtration, then the filter paper containing mycelium was placed in a 54 °C oven for drying to a constant weight. The weight difference represents the dry weight of the mycelium (Liu et al., 2019).

#### 2.8.3 HPLC detection of Wuyiencin production in the fermentation broth of overexpressed strains

Select a culture dish with well-growing *Streptomyces* spores, cut a 1 cm × 1 cm piece of the culture from the medium, and inoculate it into 100 ml of seed medium. Incubate at 220 rpm and 28 °C for 24 h. Once the seed solution becomes turbid, measure the OD_600_ value. The control group (wild-type strain) was inoculated at a 1% inoculum rate into 100 ml of Wuyiencin fermentation medium. Mutant strains were inoculated into the fermentation medium at corresponding ratios. Incubate at 220 rpm and 28 °C for 72 h. The fermentation broth was treated with a 0.22 μm bacterial filter, and the content of Wuyiencin in the fermentation broth of each strain was detected by HPLC under the following conditions (Xue et al., 2016). An Shimadzu High Performance Liquid Chromatograph LC-20A (Shimadzu Corporation, Kyoto, Japan) system was used to measure wuyiencin in the fermentation broth. Analytical conditions included the use of a SB-AQ column (4.6 mm × 250 mm, 5 μm) at a temperature of 25 °C. The mobile phase was 1.4 g/L trichloroacetic acid with a flow rate of 1 ml/min and a detection wavelength of 254 nm. The retention time for wuyiencin was 21 min (Ge et al., 2016).

#### 2.8.4 HPLC detection of toyocamycin yield in the fermentation broth of the overexpressed strain

The seed cultures of each strain were inoculated into the Toyocamycin fermentation medium under the following conditions: 28 °C, 220 rpm, and a cultivation time of 84 h. After cultivation, the Toyocamycin fermentation broth was obtained by filtering through filter paper. The content of Toyocamycin in the fermentation broth was determined using high-performance liquid chromatography (HPLC) under the following conditions (Shentu et al., 2013): Using the Shimadzu LC-20A instrument, select the C18 column (150 mm × 4.6 mm, 5 μm), methanol as mobile phase A, water as mobile phase B, perform gradient elution, flow rate 1.0 ml/min, detection wavelength 279 nm, column temperature 30 °C to detect Toyocamycin.

#### 2.8.5 Determination of fermentation and antibacterial activity of strains with overexpressed target genes

First, the tube disc method (Ma and Su, 2001) was used with red yeast as the indicator organism to determine the antibacterial activity of the fermentation broth of the CK-15 wild-type strain and the strains overexpressing each target gene. Seed solutions of the CK-15 wild-type strain and each target gene-overexpressing strain were prepared. Each strain's seed solution was inoculated into Wuyiencin fermentation medium at a 2% inoculum rate. The cultivation conditions were 28 °C, 220 rpm, with a cultivation time of 72 h. After incubation, the Wuyiencin fermentation broth was filtered through filter paper. Once the PDA medium had cooled to 37 °C, 1 ml of red yeast broth with an OD_600_ value of 1.2 was added to each 100 ml of PDA medium, mixed thoroughly, and 20 ml of the PDA medium containing red yeast broth was added to each petri dish. Place an Oxford cup on the plate and add 200 μl of fermentation broth. Set up three replicates for each treatment. After incubation at 25 °C for 48 h, observe and measure the diameter of the inhibition zone.

After determining the antibacterial activity of the fermentation broth using red yeast as the model strain, tomato gray mold fungus (*Botrytis cinerea*), wheat red mold fungus (*Gibberella sanbinetti*), apple rot fungus (*Valsa mail*), and rice blast fungus (*Magnaporthe oryzae*) were selected for antibacterial activity testing. The fermentation broth from each strain was added to PDA medium cooled to 37 °C, mixed thoroughly, and adjusted to a final concentration of 1.2% fermentation broth. After the medium solidified, sterile punch discs with a diameter of 0.6 cm were used to take samples of the aforementioned pathogenic fungi with similar growth conditions. The side with mycelium was placed face down in the center of the PDA plate. Incubate at 25 °C for 3–5 days and observe colony growth. Use PDA medium without fermentation broth as the control, with five replicates per treatment. When the control group colonies have filled the culture dish, measure the colony diameter using the cross-measurement method and calculate the antibacterial activity rate for each treatment group.

Antibacterial activity rate = (control group colony diameter – treatment group colony diameter)/control group colony diameter × 100%

#### 2.8.6 Determination of glucose content in the fermentation broth of strains with overexpressed target genes

Seed solutions of each strain were inoculated into Wuyiencin fermentation medium at a specified ratio, incubated at 28 °C and 220 rpm for 5 days, with samples collected every 12 h, totaling 10 samples, with three replicates per treatment. Glucose content in the fermentation broth at different time points was detected using a glucose content detection kit (Beijing Box Biotechnology, Beijing, China), with the detection method as follows:

(1) Dilute the standard solution and plot a standard curve. Refer to Table 1.

**Table 1:** Preparation of glucose standard curve solution.

Sequence number	A	1	2	3	4	5	6
Concentration before dilution (mg/ml)	10	1	1	1	1	1	1
Standard liquid volume (μl)	100	150	100	100	150	100	50
Volume of distilled water (μl)	900	100	100	150	350	400	450
Diluted concentration (mg/ml)	1	0.6	0.5	0.4	0.3	0.2	0.1

#### 2.8.7 Determination of ammonia nitrogen content in the fermentation broth of strains with overexpression of target genes

Determination of ammonia nitrogen content in fermentation broth of different strains at different time points using the nitroprusside method.

(1) Preparation of the test solution: dissolve 35 g of phenol and 0.2 g of sodium nitroprusside thoroughly in distilled water, then dilute to 1 L with distilled water and mix thoroughly to obtain Solution A. Dissolve 18 g of NaOH in distilled water, add 1.8 ml of 0.04 M sodium hypochlorite solution, and dilute with distilled water to 1 L. Mix thoroughly to obtain Solution B. Solution B is prone to degradation and decomposition, so it should be prepared fresh as needed.
(2) Take 100 μl of each sample and dilute with distilled water to 4 ml.
(3) Take 1 ml of each diluted sample into a glass test tube, add 5 ml of Solution A and 5 ml of Solution B in sequence, mix thoroughly, and incubate at 37 °C for 35 min. Each treatment is set with three replicates. For the control group, replace the diluted fermentation broth with distilled water.
(4) Preheat the spectrophotometer SpectraMax M5e (Molecular Devices, Sunnyvale, CA, USA) for 30 min and adjust the wavelength to 625 nm. Zero the instrument using the control group, then measure the absorbance values of each sample.
(5) Substitute the measured absorbance values into the formula: Ammonia nitrogen content = (0.2004 × absorbance value – 0.0018) × sample dilution factor × 0.212 to determine the ammonia nitrogen content of each sample.

## 3 Results and conclusions

### 3.1 Differential gene analysis between Δ*toyG* and wild-type CK-15

**Figure 1:**
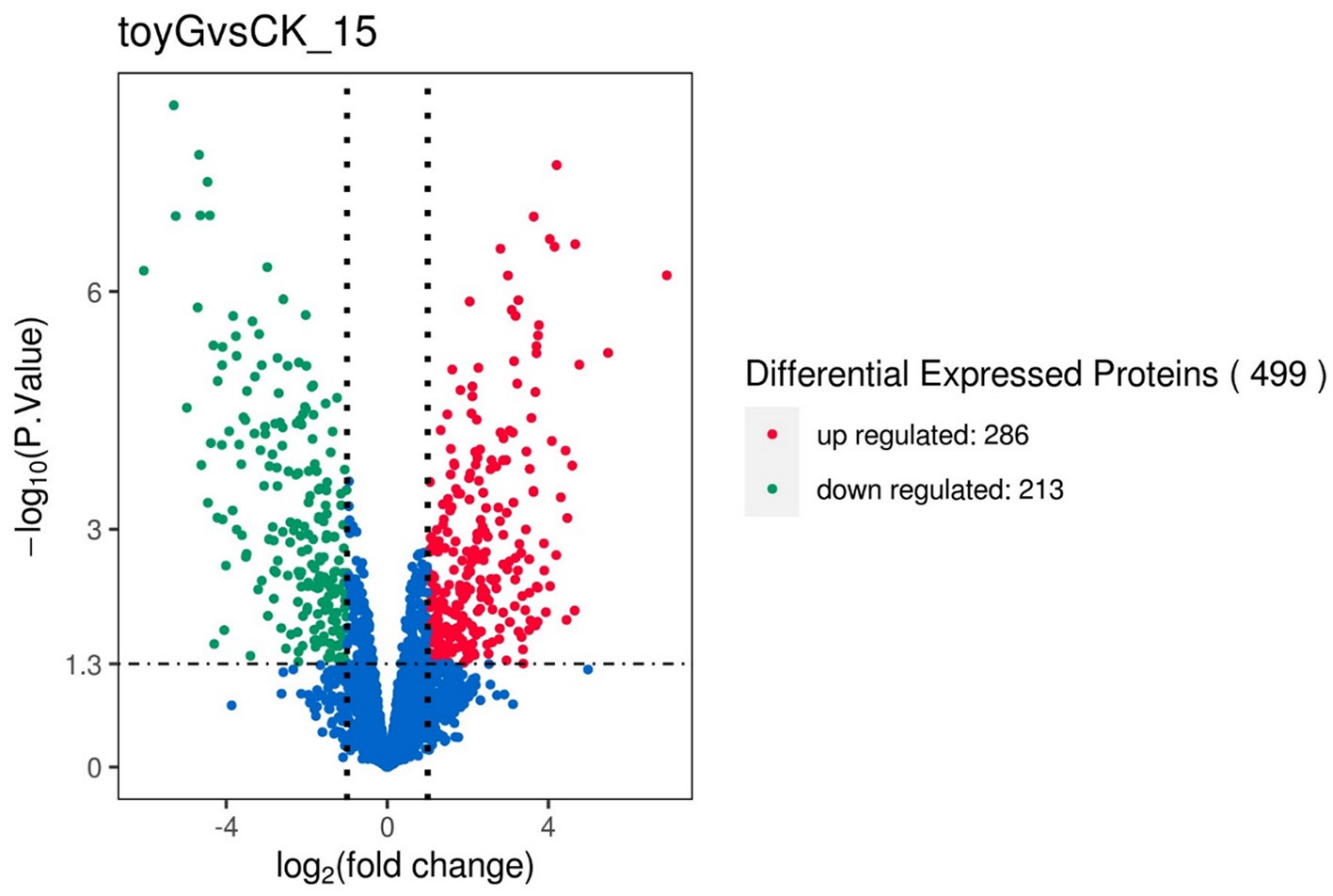
Δ*toyG* vs. CK-15 group differential protein volcano map. Transcriptome sequencing was performed with three biological replicates per strain (*n* = 3), where *n* represents the number of independent RNA extraction samples from wild-type CK-15 and Δ*toyG* mutant strains.

Based on |log2FoldChange| > 1, *P*adj < 0.05, and readcount >60, differentially expressed genes were screened. Compared with the CK-15 control group (Figure 1), the Δ*toyG* group had a total of 1,038 differentially expressed genes, including 545 up-regulated genes and 493 down-regulated genes. Functional annotation of the differentially expressed genes revealed that the up-regulated genes in the Δ*toyG* group were mainly involved in phosphoribosyl pyrophosphate kinase, HAD hydrolase-like proteins, and glycosyltransferases; The functions of the downregulated differentially expressed genes in the Δ*toyG* group were concentrated in adenosine succinate synthase (*toyG*), succinate dehydrogenase cytochrome b subunit, TerB family tellurite resistance protein, and acylneuraminic acid cytidine transferase (Figure 1).

### 3.2 GO functional and KEGG metabolic pathway analysis of differentially expressed genes

**Figure 2:**
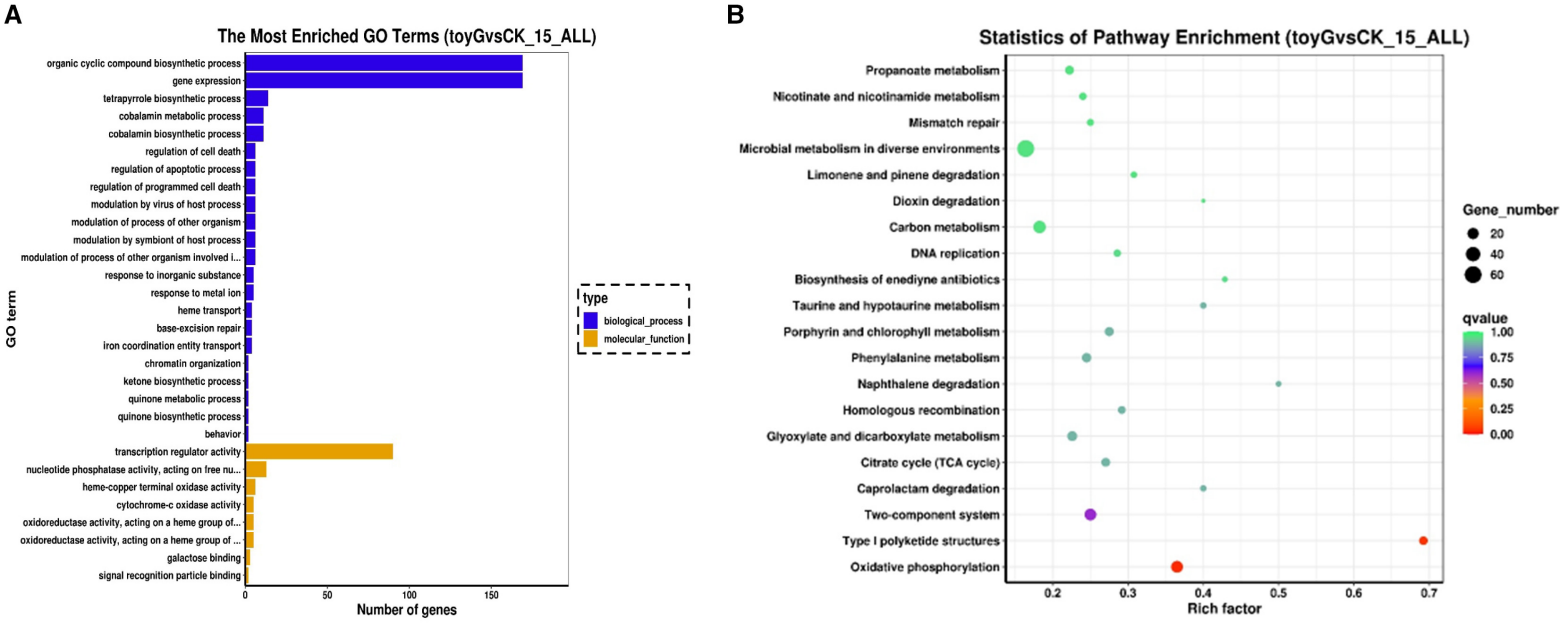
GO terms and KEGG pathways enriched in Δ*toyG* group differentially expressed genes. **(A)** Top 30 GO terms enriched in Δ*toyG* group differentially expressed genes. **(B)** Top 30 KEGG pathways enriched in Δ*toyG* group differentially expressed genes. All enrichment analyses were based on transcriptome data with three biological replicates per strain (*n* = 3), where *n* represents the number of independent RNA extraction samples.

Perform GO enrichment analysis on the differentially expressed genes screened from the Δ*toyG* group. The top 30 GO terms enriched in the 1,038 differentially expressed genes from the Δ*toyG* group are listed below. The top five enriched GO terms and the number of differentially expressed genes they contain are as follows: six genes related to heme copper terminal oxidase activity, 11 differentially expressed genes involved in cyanocobalamin metabolism, 11 differentially expressed genes involved in cyanocobalamin biosynthesis, five differentially expressed genes involved in cytochrome C oxidase activity, five differentially expressed genes involved in oxidoreductase activity, and five differentially expressed genes involved in the heme donor group (Figure 2A). The above results indicate that significantly differentially expressed genes are primarily enriched in biological processes and molecular functions that influence the synthesis of secondary metabolites.

KEGG enrichment analysis of the differentially expressed genes in the Δ*toyG* group revealed that the top five pathways were oxidative phosphorylation, type I polyketide biosynthesis, two-component systems, porphyrin and chlorophyll metabolism, and the tricarboxylic acid cycle, with 23, 9, 23, 11, and 10 differentially expressed genes enriched in each pathway, respectively (Figure 2B). In summary, the *toyG* gene primarily influences substrate supply, PKS-related gene expression, and the activation of key enzymes during the synthesis of secondary metabolites, thereby affecting metabolic pathway flow and altering the synthesis pathways of secondary metabolites.

### 3.3 Selection of target genes

Based on transcriptomic analysis of *S. noursei* Δ*toyG* and CK-15 wild-type strains, a total of 1,038 differentially expressed genes were identified, and KEGG enrichment analysis revealed these genes were primarily enriched in metabolic pathways such as oxidative phosphorylation, polyketide biosynthesis, and two-component systems. Given that the core research focus is to clarify the regulatory mechanism of nucleoside-type secondary metabolites (wuyiencin and toyocamycin)—whose synthesis is disrupted in the Δ*toyG* mutant due to impaired nucleoside metabolism—we prioritized screening differential genes closely associated with this metabolic process.

Among the differentially expressed genes, SE95_RS25840 encodes a histidine kinase (SHK), which, along with the response regulator protein, constitutes the two components of a two-component system. When an organism receives external environmental signals, it triggers self-phosphorylation of the histidine kinase; the phosphorylated histidine kinase then interacts with the response regulator protein, inducing its phosphorylation to regulate the expression of downstream genes (Stock et al., 2000). Three key lines of evidence further support the rationality of selecting SE95_RS25840 *(SHK*) over other differential genes: first, the two-component system to which SHK belongs is the core pathway for transducing metabolic signals and regulating secondary metabolism in prokaryotes—a function highly relevant to our focus on nucleoside metabolism—whereas differentially expressed genes in pathways such as oxidative phosphorylation (primarily involved in energy production) and polyketide biosynthesis (associated with non-nucleoside secondary metabolites) have no direct link to nucleoside synthesis, making them irrelevant to the study's target. Second, SHK is coexpressed with the core metabolic defect of the Δ*toyG* mutant (abnormal purine metabolism, manifested by disrupted wuyiencin and toyocamycin production) and directly integrated into the purine nucleoside metabolic network; in contrast, other differentially expressed two-component system genes are enriched in pathways unrelated to nucleoside metabolism (e.g., environmental stress response) and thus excluded from priority validation. Third, SHK exhibits conserved homologous functions: its homologs in *S. coelicolor* and *Streptomyces avermitilis* have been confirmed to modulate secondary metabolism by regulating nitrogen metabolism further validating its relevance to nucleoside metabolism regulation.

**Figure 3:**
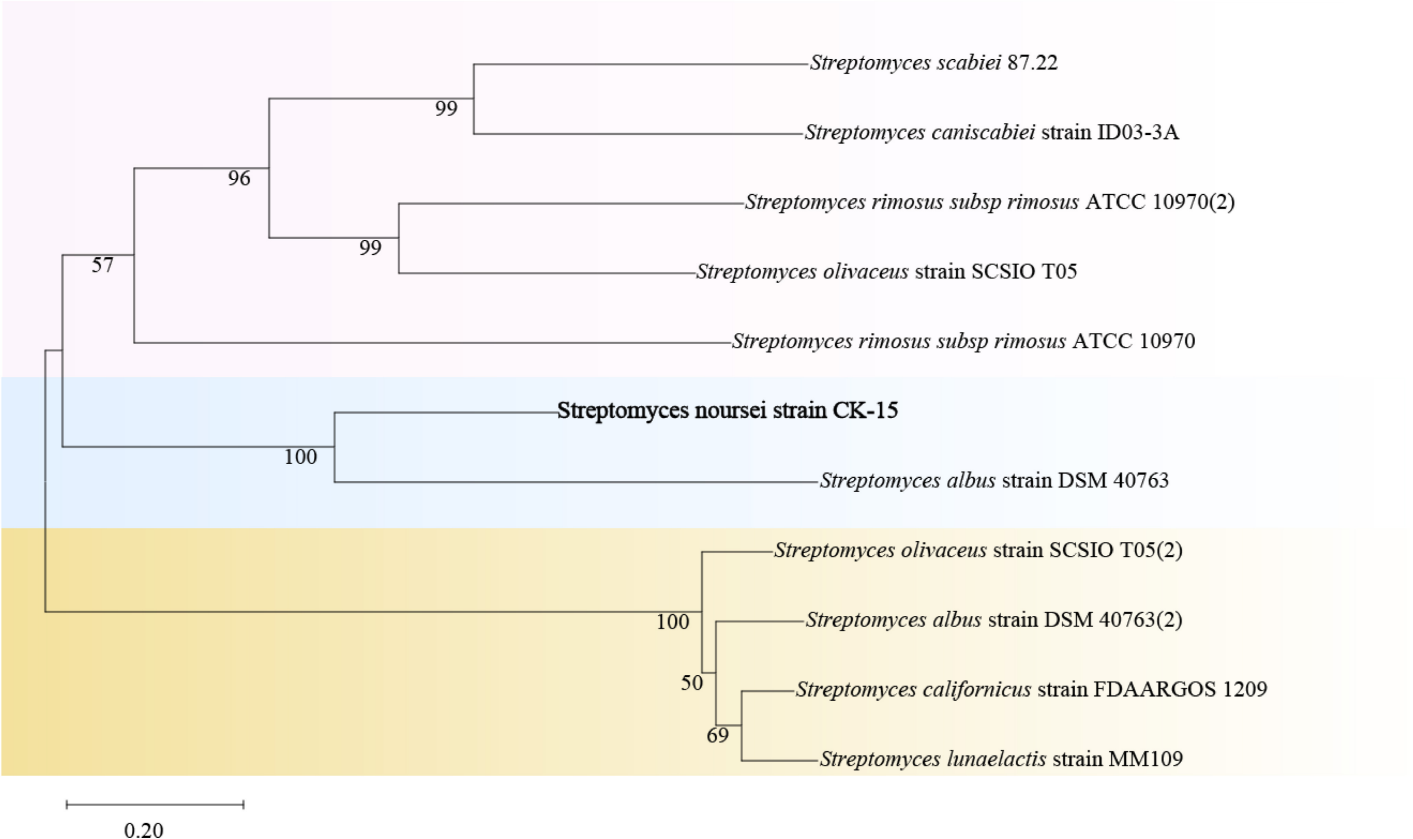
Phylogenetic NJ tree based on the sequence of the encoded histidine kinase homologous gene.

Therefore, the differential gene SE95_RS25840 (SHK) was selected for functional validation to elucidate its impact on the synthesis of secondary metabolites in *S. noursei* CK-15. To further explore the function of histidine kinase in *S. noursei* CK-15, the NJ method was used to construct a phylogenetic tree of histidine kinases from 11 *Streptomyces* strains. The results showed that the histidine kinase in CK-15 shares a certain degree of phylogenetic affinity with histidine kinases in most *Streptomyces* strains. Specifically, the branch node connecting CK-15 and *Streptomyces albus* strain DSM 40763 is closely linked, with a higher phylogenetic affinity than other *Streptomyces* strains (Figure 3).

### 3.4 Construction and screening of *OSHK* mutant strains

PCR amplification was performed according to the described method to obtain the target gene *SHK* (1,308 bp), which was then integrated into the vector pSETC to obtain the recombinant plasmid pSETC-*SHK*. The recombinant plasmid was transferred into the CK-15 strain via conjugation, and strains with ampicillin resistance were screened to successfully obtain the overexpression strain *oSHK* (Supplementary Figure S1).

### 3.5 Validation of *SHK* gene expression in overexpressed strains by quantitative real-time PCR (qPCR)

**Figure 4:**
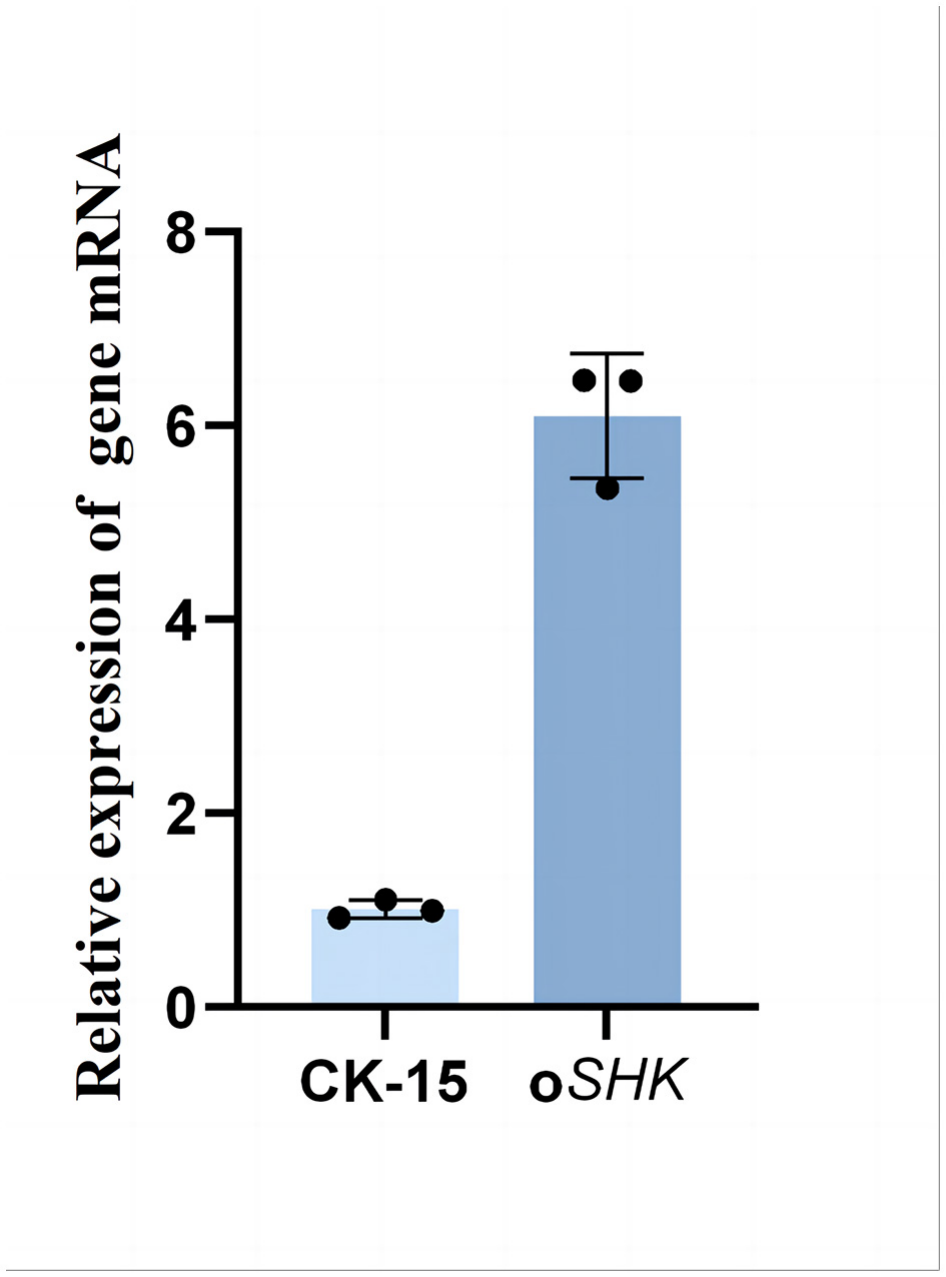
Relative mRNA transcription level of the SHK gene in wild-type strain CK-15 and SHK-overexpressing strain oSHK. The relative expression level of the SHK gene was quantified using the 2-ΔΔCtmethod, with 16S rRNA employed as the reference gene. Data are presented as mean ± standard deviation, where *n* = 3 biological replicates (each replicate corresponds to independent bacterial cultivation, total RNA extraction, cDNA synthesis, and qPCR assays).

Following the aforementioned method, total RNA was isolated from *S. noursei* CK-15 and *S. noursei oSHK*, and the integrity of the isolated RNA was verified by PCR—with the results shown in Supplementary Figure S2, where distinct 16S rRNA and 23S rRNA bands were observed, indicating qualified RNA integrity. Subsequently, Total RNA was extracted from both strains and reverse-transcribed into complementary DNA (cDNA), which was then used as the template for quantitative real-time PCR (qPCR) to validate the expression level of the SHK gene in the oSHK strain (and the wild-type CK-15 strain as the control). 16S rRNA was employed as the reference gene to normalize for variations in RNA reverse transcription yield across different samples. The relative expression level of the SHK gene was quantified using the 2^(−Δ*Ct*)^ method for relative quantification. The results, presented in Figure 4, demonstrated that the expression level of the SHK gene in the oSHK strain is approximately sixfold higher than that in the wild-type strain CK-15.

### 3.6 Analysis of growth characteristics of *OSHK* strains

**Figure 5:**
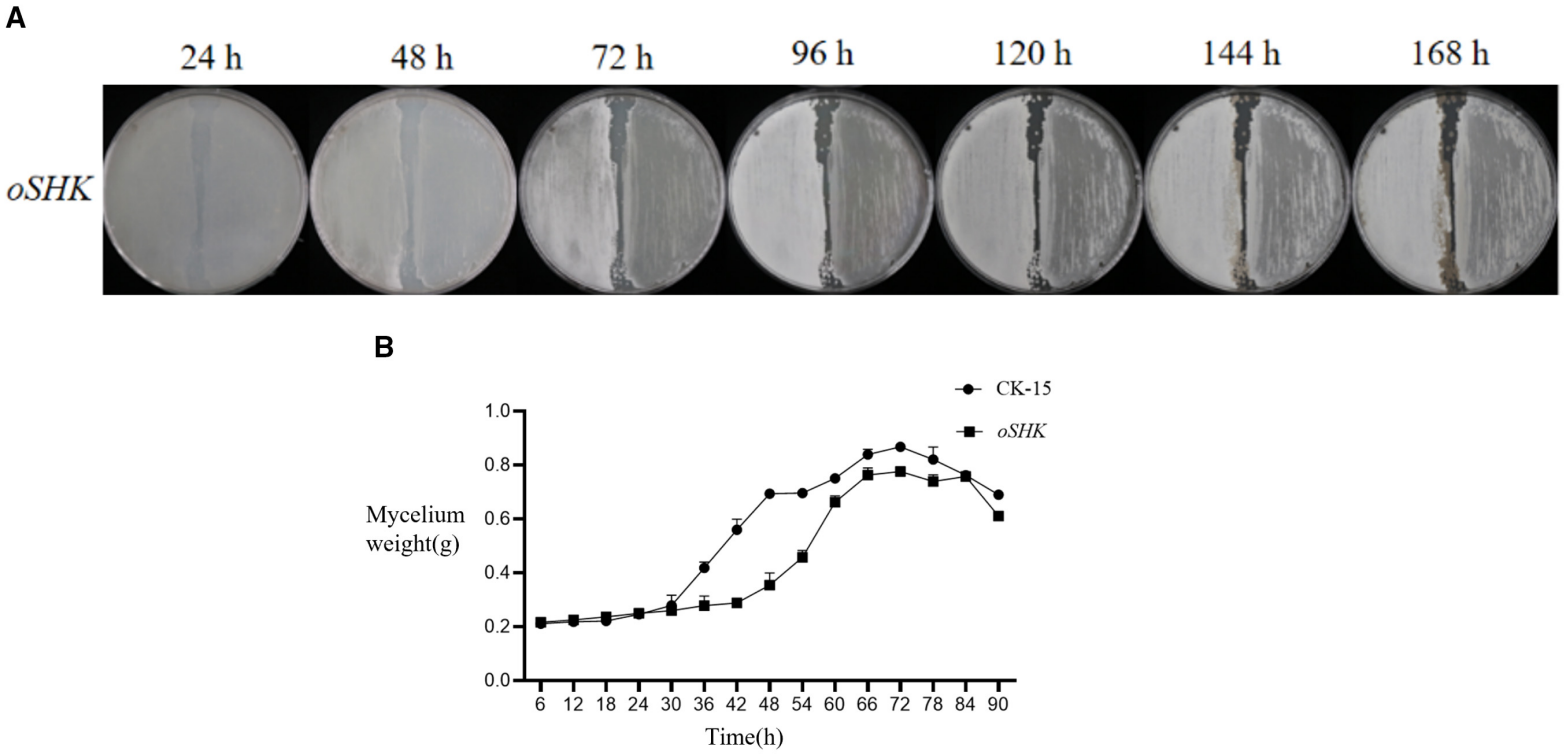
Colony growth phenotypes and biomass indicators of *oSHK*. **(A)** Growth of oSHK strain on plates at different time points (*n* = 3 biological replicates, where *n* represents the number of independent plate streaking experiments). **(B)** Curve showing changes in dry weight of mycelium of the oSHK strain at different time intervals. Mycelium dry weight was measured with three biological replicates per time point (*n* = 3).

First, the CK-15 wild-type strain and the *SHK* gene-overexpressing mutant strain were streaked onto the same MS medium. The left side of the plate was CK-15, and the right side was the *SHK* gene-overexpressing mutant strain. Observations were made every 24 h. The experimental results are shown in Figure 5A: Starting from 72 h, the *oSHK* strain exhibited poorer growth compared to the CK-15 wild-type strain, with uneven mycelial growth on the plate. Therefore, the *SHK* gene inhibits the mycelial growth and spore production rate of the strain.

Next, the dry weight of mycelium at different time points was measured for both the CK-15 wild-type strain and the *SHK* gene-overexpressing mutant strain. The experimental results indicate (Figure 5B) that the *oSHK* strain significantly inhibits mycelium growth compared to CK-15. Between 0 and 30 h, there was no significant difference in mycelium dry weight between the two strains. However, starting from 30 h, the mycelium dry weight of the *oSHK* strain was significantly lower than that of the CK-15 wild-type strain. At 48 h, the mycelium dry weight of the CK-15 strain was 0.34 g higher than that of the *oSHK* mutant strain. This indicates that the overexpression of the *SHK* gene inhibits mycelium growth in the strain.

### 3.7 Detection of secondary metabolites of *OSHK* mutant strains

**Figure 6:**
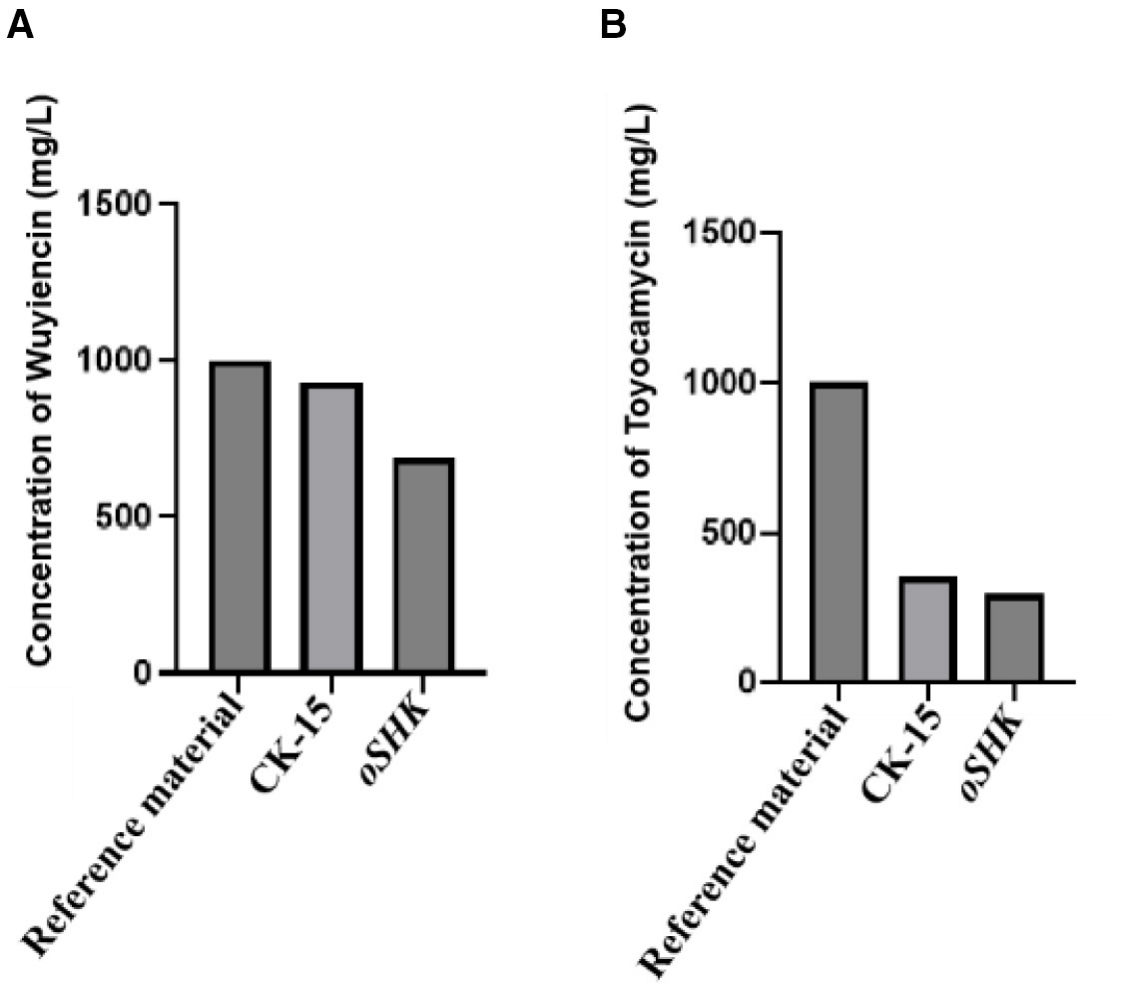
Determination of secondary metabolite content in the fermentation broth of the *oSHK* strain. **(A)** Determination of Wuyiencin content. **(B)** Determination of Toyocamycin content. HPLC detection was performed with three biological replicates per strain (*n* = 3), where *n* represents the number of independent fermentation experiments.

The content of Wuyiencin and Toyocamycin in the fermentation broth of the *oSHK* was determined by HPLC. As shown in Figure 6, the concentration of Wuyiencin in the *oSHK* strain was 689.46 mg/L, a 25.94% reduction compared to the wild-type CK-15 (Figure 6A); meanwhile, the Toyocamycin content was 293.79 mg/L, a 17.64% decrease relative to the wild-type (Figure 6B). Importantly, the two metabolites exhibited a consistent downward trend in the *oSHK* strain, with no significant increase in either metabolite accompanying the decrease of the other. This synchronous change differs from the “competitive regulation” pattern, where metabolic flux is redirected to one metabolite at the expense of the other) and provides preliminary evidence that *SHK* may regulate the biosynthesis of Wuyiencin and Toyocamycin through a coordinated mechanism.

### 3.8 Determination of glucose content in the fermentation broth of *OSHK* mutant strains

**Figure 7:**
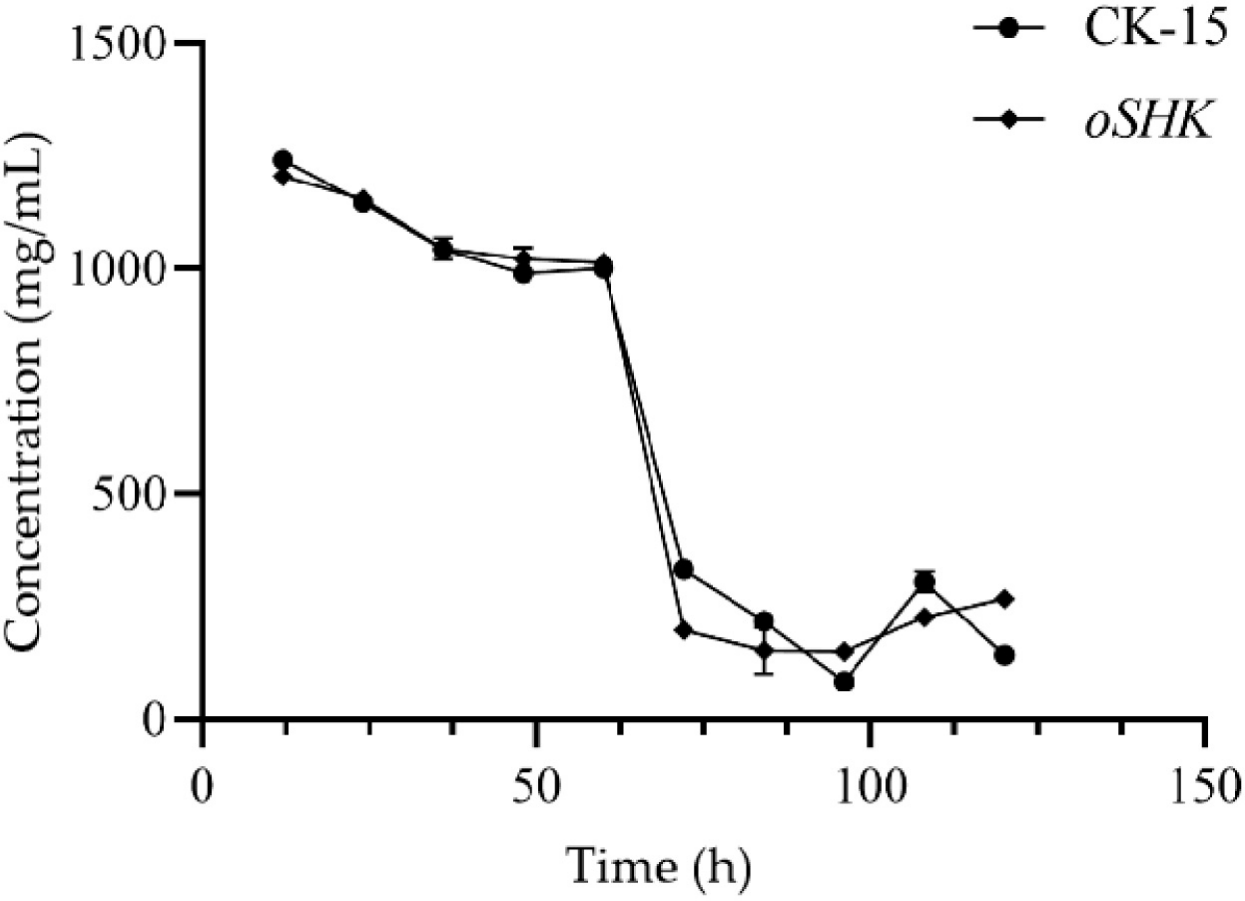
Determination of glucose content in the fermentation broth of the *oSHK* strain. Glucose content was detected using a glucose assay kit with three biological replicates per time point (*n* = 3), where *n* represents the number of independent fermentation broth samples.

Since the fermentation medium formulation for Wuyiencin contains 20 g/L of glucose, the glucose content in the fermentation broth reaches its maximum value at 12 h. During fermentation, the strain utilizes glucose for metabolism, thereby producing secondary metabolites. Over time, the glucose content in the fermentation broth of the CK-15 wild-type strain exhibits a trend of first decreasing, then increasing (Figure 7), and then decreasing again, with a sharp drop in glucose content between 60 and 72 h. Compared to the CK-15 wild-type strain, there is no significant difference in glucose content changes in the *oSHK* strain. However, between 96 and 120 h, the glucose content in the fermentation broth of the CK-15 wild-type strain showed a trend of first increasing and then decreasing, while the glucose content in the fermentation broth of the *oSHK* strain showed an increasing trend.

### 3.9 Determination of ammonia and nitrogen content in fermentation broth of *OSHK* mutant strain

**Figure 8:**
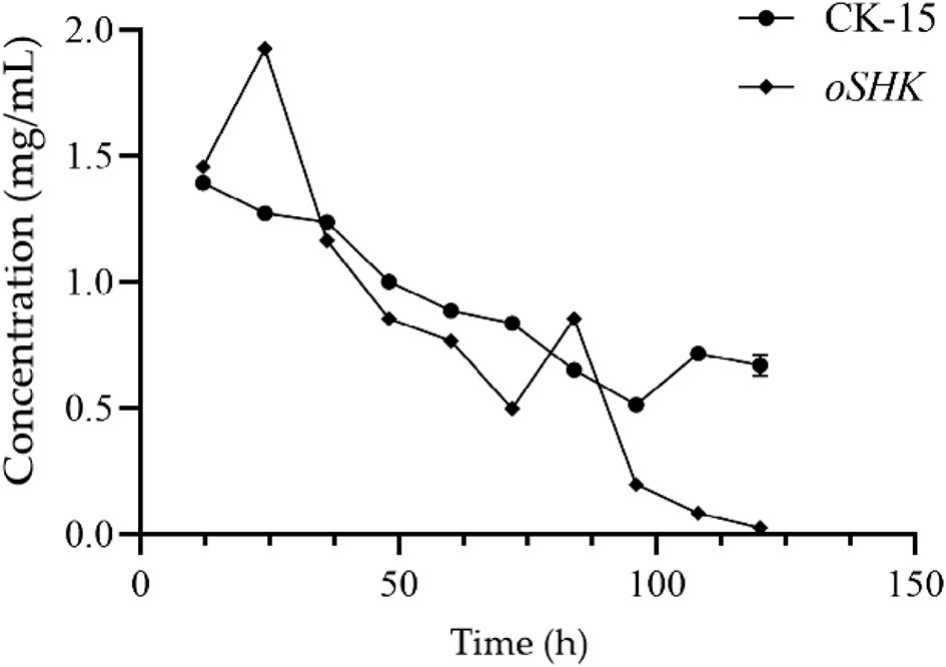
Determination of ammonia nitrogen content in the fermentation broth of the *oSHK* strain. Ammonia nitrogen content was measured via the nitroprusside method with three biological replicates per time point (*n* = 3), where *n* represents the number of independent fermentation broth samples.

Using the nitroprusside method, the ammonia nitrogen content in the fermentation broth of different strains at different time points was measured. As the fermentation time increased, the ammonia nitrogen content in the fermentation broth showed an overall downward trend (Figure 8). Throughout the fermentation process, the ammonia nitrogen content in the fermentation broth of the wild-type strain CK-15 showed a steady decreasing trend. After overexpression of the *SHK* gene, the ammonia nitrogen content in the fermentation broth of the strain exhibited significant instability compared to the wild-type strain CK-15, frequently exhibiting abrupt increases and decreases in ammonia nitrogen content throughout the fermentation process. This is analyzed to be due to the overexpression of the *SHK* gene interfering with the strain's utilization of nitrogen sources, thereby affecting the synthesis of secondary metabolites in the strain.

### 3.10 Determination of fermentation and antibacterial activity of *OSHK* mutant strain

**Figure 9:**
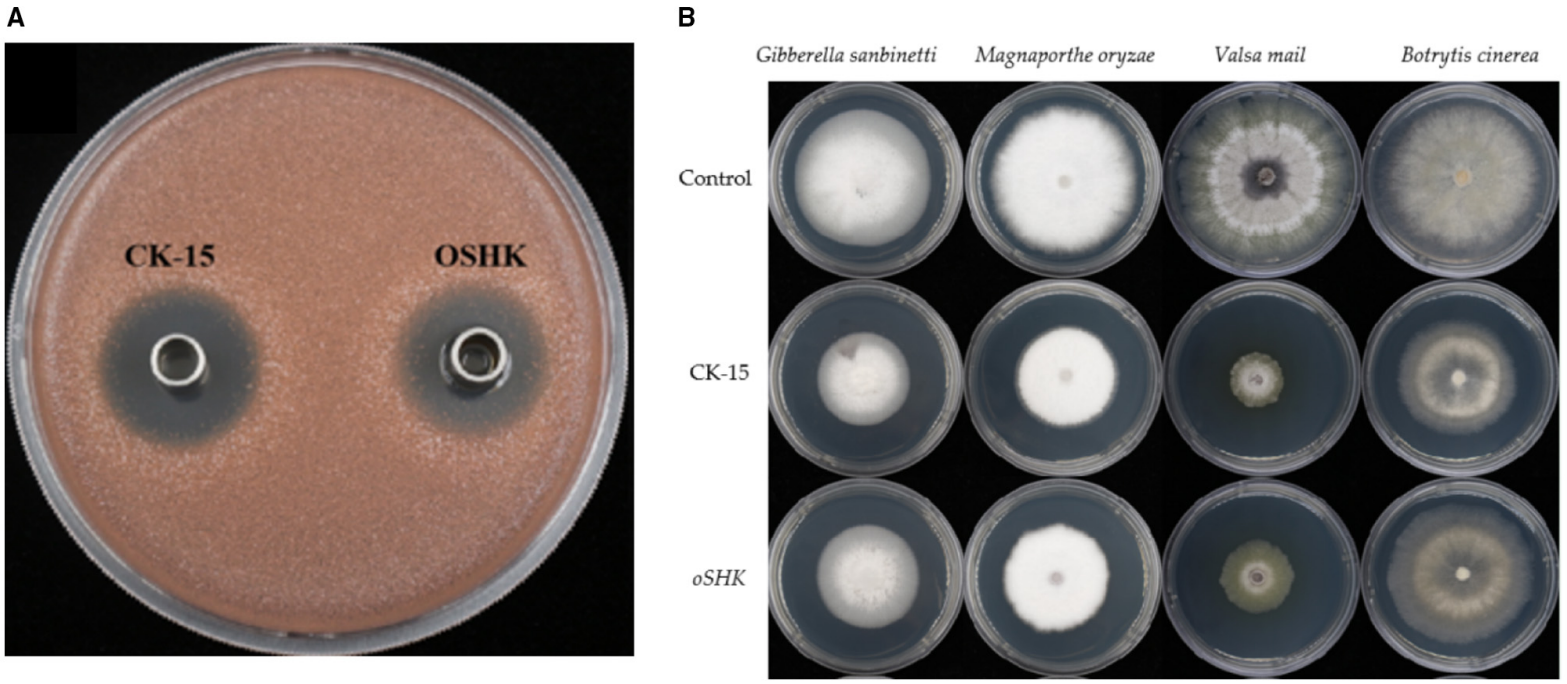
Antimicrobial activity of *oSHK* strain fermentation broth and its antimicrobial effect on four pathogenic bacteria. **(A)** Determination of antimicrobial activity of *oSHK* strain fermentation broth (inhibition zone diameter). The tube disc method was used with three biological replicates per strain (*n* = 3), where *n* represents the number of independent inhibition zone assays. **(B)** Antimicrobial effect of *oSHK* strain fermentation broth on four pathogenic fungi. Colony diameter was measured with five biological replicates per pathogen (*n* = 5).

First, the antibacterial activity of the mutant strain fermentation broth against red yeast was tested. After triplicate measurements, the antibacterial diameter of the *oSHK* strain was approximately 9.09% smaller than that of the wild-type CK-15 (Figure 9A). To correlate this activity change with the content of known antibacterial metabolites, we compared the magnitude of antibacterial activity reduction with the decrease in Wuyiencin and Toyocamycin yields: the oSHK strain exhibited a 25.94% reduction in Wuyiencin and a 17.64% reduction in Toyocamycin, while the antibacterial diameter against red yeast decreased by only 9.09%. The reduction in antibacterial activity was smaller than the individual decreases in either metabolite—suggests two possibilities: (1) Wuyiencin and Toyocamycin may exert synergistic antibacterial effects against, such that their synchronous reduction leads to a less-than-additive decline in overall activity; (2) the fermentation broth may contain other unquantified antibacterial compounds whose production was not affected by SHK overexpression, partially compensating for the loss of activity from Wuyiencin and Toyocamycin.

Next, the antibacterial effect of the mutant strain fermentation broth against pathogenic fungi was tested using the mycelial growth rate method (Figure 9B; Table 2). Consistent with the red yeast assay, the antimicrobial efficacy of the oSHK strain was lower than that of CK-15. This phenomenon is primarily attributed to the decreased content of Wuyiencin and Toyocamycin.

**Table 2:** Antifungal effects of *oSHK* strain fermentation broth on four pathogenic fungi.

Pathogenic bacteria	Bacterial strain	Colony diameter (cm)	Inhibition ratio (%)
*Botrytis cinerea*	Control	8.33 ± 0.12a	–
	CK-15	5.93 ± 0.29c	28.8
	*oSHK*	6.40 ± 0.20b	23.2
*Valsa mail*	Control	8.10 ± 0.10a	–
	CK-15	2.83 ± 0.24c	65.02
	*oSHK*	3.60 ± 0.44b	55.56
*Magnaporthe oryzae*	Control	6.92 ± 0.76a	–
	CK-15	4.60 ± 0.20c	33.49
	*oSHK*	5.05 ± 0.87b	26.99
*Gibberella sanbinetti*	Control	6.31 ± 0.14a	–
	CK-15	4.35 ± 0.50c	31.13
	*oSHK*	4.87 ± 0.76b	22.96

The different letters (abc) indicate significant differences.

Histidine kinases are essential elements in bacteria for detecting stimuli from the environment and modulating physiological processes. The advancement of molecular biology, structural biology, and synthetic biology has improved research into their functions and mechanisms (Kansari et al., 2024; Pérez-García et al., 2016). This study involved the creation of an overexpressing strain of *S. noursei oSHK* to examine the role of the *SHK* gene. Dynamic detection of ammonia nitrogen content in the fermentation broth revealed significant fluctuations in the oSHK strain compared to the wild-type CK-15, which provides indirect evidence that overexpression of *SHK* may interfere with the strain's nitrogen source utilization. Nitrogen metabolism in microbial regulatory networks is tightly governed by the ntr system (Bascarán et al., 1989). Based on this, we propose a hypothetical mechanism: *SHK* may disrupt the phosphorylation cascade of the two-component regulatory system or competitively bind to PII proteins, thereby impairing nitrogen signal transduction.

The study revealed that the overexpression of the *SHK* gene resulted in a diminished spore production rate and suboptimal growth conditions in the *S. noursei oSHK* mutant strain. The cell cycle and differentiation are essential for bacterial growth, reproduction, and morphological development. Tan and Chater (1993) discovered that the *whiG* gene in *S. coelicolor* produces the σ^WhiG^ factor, which identifies promoters with the conserved CCGA pattern and initiates the transcription of genes associated with spore production. High-copy whiG-dependent promoters impede spore production in aerial hyphae by competitively binding σ^WhiG^, hence affirming their function in beginning spore formation via gene expression control. Aínsa et al. (2000) found the *whiA* gene in *S. coelicolor*, which encodes a protein conserved in Gram-positive bacteria and is crucial *for* spore formation. A deletion leads to elongated, coiled aerial hyphae devoid of spore septa, inhibiting spore chain development. This gene has elevated expression during spore formation through an autoregulatory promoter, synergistically coordinating signals with σ^WhiG^ to modulate gene expression associated with septum formation and spore maturation. *SHK* overexpression may disrupt differentiation regulation by reducing the activity of transcription factors linked to spore formation or obstructing the binding of critical genes for spore formation to their target genes. This causes a disruption of the cell cycle (incongruence between nutrient growth and spore differentiation timing), ultimately leading to growth retardation and problems in spore generation.

This study found that the overexpression of histidine kinase decreased the output of secondary metabolites. Lu et al. (2011) discovered that particular regulatory factors and precursor synthesis genes directly govern critical stages in antibiotic synthesis by negatively inhibiting pathways, hence enhancing yield. Bibb (2005) indicated that histidine kinases in *Streptomyces* convey signals via a phosphorylation cascade to modulate secondary metabolic gene clusters, and that the two-component system can universally govern various antibiotic production pathways. Tian et al. (2020) employed histidine kinase-associated quorum sensing elements in the EQCi system to progressively modulate the distribution of primary and secondary metabolism, facilitating bacterial growth while concurrently triggering the synthesis of secondary metabolites as needed, thus enhancing yield. Consequently, histidine kinase in CK-15 can affect secondary metabolite production by signal transduction, precise control of essential genes, and the integration of environmental inputs.

In summary, overexpression of the *SHK* gene may interfere with nitrogen utilization in *S. noursei* CK-15, and this phenomenon is indirectly supported by the unstable ammonia nitrogen content in the fermentation broth. A hypothetical mechanism is proposed: this interference may be mediated by SHK affecting the phosphorylation cascade of the two-component system or competing for binding with PII proteins, thereby blocking nitrogen signal transduction associated with the ntr system. However, this mechanism requires further validation through targeted experiments, such as: verifying the interaction between *SHK* and PII proteins using techniques including yeast two-hybrid (Y2H), co-immunoprecipitation (Co-IP), or surface plasmon resonance (SPR); and detecting the phosphorylation levels of key components of the ntr system in both the *oSHK* mutant strain and the wild-type strain to clarify whether overexpression of *SHK* affects the phosphorylation cascade of the ntr system. Additionally, *SHK* overexpression leads to reduced spore production rates and impaired growth in the strain, potentially by inhibiting the activity of transcription factors associated with spore formation or blocking the binding of essential genes, thereby disrupting the coordinated regulation of differentiation processes by other related genes and causing cell cycle disorders. Furthermore, overexpression of histidine kinase reduces the yield of secondary metabolites, confirming its mechanism of influencing secondary metabolism through signal transduction, targeted regulation, and environmental signal integration, thereby comprehensively inhibiting the growth and secondary metabolite synthesis of CK-15.

Our results demonstrate that the *SHK* gene modulates the biosynthesis of the Wuyiencin and Toyocamycin nucleoside-type secondary metabolites in a synergistic rather than competitive manner, and this conclusion is supported by three lines of experimental evidence: First, in the *oSHK*, the yields of both Wuyiencin and Toyocamycin were significantly reduced compared to the wild-type CK-15 Wuyiencin content decreased by 25.94% and Toyocamycin content decreased by 17.64%. Notably, there was no trade-off phenomenon (one metabolite increasing while the other decreasing) which is typical of competitive regulation; instead, the two metabolites exhibited a consistent downward trend, indicating that *SHK* does not regulate their biosynthesis through mutually exclusive pathways. Second, KEGG enrichment analysis of the Δ*toyG* mutant revealed that differentially expressed genes (including *SHK*) were enriched in the two-component system and nucleoside metabolism-related pathways. Since both Wuyiencin and Toyocamycin belong to nucleoside-type metabolites and their biosynthesis relies on common upstream metabolic intermediates, *SHK* is inferred to modulate the two metabolites by regulating a shared upstream metabolic network (e.g., nitrogen signal transduction or nucleoside precursor supply) rather than acting independently on their respective biosynthetic pathways. Third, the antibacterial activity assay showed that the fermentation broth of the *oSHK* strain exhibited significantly reduced inhibitory effects against *Rhodotorula rubra* and four plant pathogenic fungi compared to the wild-type. This functional phenotype is consistent with the synchronous reduction of Wuyiencin and Toyocamycin—both of which contribute to the strain's antibacterial activity—further confirming that *SHK* regulates the two metabolites in a coordinated manner to maintain the strain's biological function. In contrast, “independent regulation” would imply that *SHK* acts on distinct, non-overlapping pathways for Wuyiencin and Toyocamycin biosynthesis, without consistent changes in their yields or associated functional phenotypes. The above evidence thus excludes independent regulation and supports a synergistic regulatory role of *SHK*.

Additionally, the reduction in antibacterial activity of the *oSHK* strain against *R. rubra* was smaller than the individual decreases in Wuyiencin and Toyocamycin, suggesting synergistic antibacterial effects between the two metabolites. This quantitative discrepancy also implies that the fermentation broth may contain unquantified antibacterial compounds whose production is not regulated by *SHK*, which partially compensates for the activity loss from Wuyiencin and Toyocamycin. Future non-targeted metabolomics studies are needed to validate the presence of these unquantified compounds and clarify their contribution to the strain's antibacterial activity.

This study performed just initial functional confirmation of the *SHK* gene by creating an overexpressing strain. Additional investigation into the function of the *SHK* gene can be conducted by creating gene knockout mutant strains, thereby elucidating the role of histidine kinase in *S. noursei* CK-15. Future research may find more genes to clarify their regulation mechanisms, providing theoretical advice for the enhancement of Wuyiencin synthesis.

## Data Availability

The GAS number of this data is: CRA039805. The relevant link is as follows: https://ngdc.cncb.ac.cn/gsub/submit/gsa/subCRA065321/finishedOverview.
