# Supplementary material for: The regulatory role of histidine kinase in modulating nucleoside metabolites in Streptomyces noursei CK-15
Source: Front Microbiol. 2026 Apr 1;17:1692886. doi: 10.3389/fmicb.2026.1692886 (PMC13079031; doi:10.3389/fmicb.2026.1692886)
Supplement: Supplementary file 1 [file Supplementary_file_1.docx]

Supplementary Materials

**
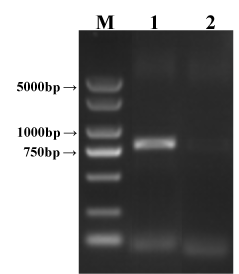
**

**Figure S1**. PCR amplification of the target gene overexpression combined with the Apr fragment, where M: DL5000 marker, 1 is pSETC-*SHK*, and 2 is the negative control CK-15.

**Table S1** Primer sequences used in this study

| Primer Name | Sequence (5'-3') | Product Size | Application |
| --- | --- | --- | --- |
| Apra-F | GAGTGCAATGTCGTGCAATACGA | 777bp | Amplification of the apramycin resistance gene |
| Apra-R | GCATTCTTCGCATCCCGCCT |  |  |
| *SHK*-F | TCAATACTCGATTAGGGATCCATGAGACAGGTGCAGCCGAA | 1266bp | Amplification of the target sequence SHK |
| *SHK*-R | CACGTCTTAAGACGTACTAGTCTAGGCCGGCCCGTTCAC |  |  |

**Table S2** Antibiotic concentrations used in this study

| Antibiotic name | Applicable strains | Concentration(μg/mL) |
| --- | --- | --- |
| apramycin | *E. coli*DH5α、*E. coli* ET12567/*pUZ8002*、pSETC  Streptomyces conjugates | 50 |
| chloromycetin | *E. coli* ET12567/*pUZ8002* | 50 |
| kanamycin | *E. coli* ET12567/*pUZ8002* | 25 |
| nalidixic acid | Streptomyces conjugates | 50 |

**Table S3** Real-time fluorescence quantitative PCR amplification system

| component | volume |
| --- | --- |
| ArtiCan^CEO^ SYBR qPCR Mix | 10 μL |
| 10 μM Primer F | 0.8 μL |
| 10 μM Primer R | 0.8 μL |
| Template（cDNA） | 1 μL |
| ddH_2_O | 7.4 μL |
| Total | 20 μL |

**Table S4** List of Instruments and Equipment Used in the Study

| Equipment Name | Manufacturer | Country |
| --- | --- | --- |
| apramycin | *E. coli*DH5α、*E. coli* ET12567/*pUZ8002*、pSETC  Streptomyces conjugates | 50 |
| chloromycetin | *E. coli* ET12567/*pUZ8002* | 50 |
| kanamycin | *E. coli* ET12567/*pUZ8002* | 25 |
| nalidixic acid | Streptomyces conjugates | 50 |
